# Sample Size Requirements for Estimation of Item Parameters in the Multidimensional Graded Response Model

**DOI:** 10.3389/fpsyg.2016.00109

**Published:** 2016-02-09

**Authors:** Shengyu Jiang, Chun Wang, David J Weiss

**Affiliations:** Psychology, University of Minnesota Twin CitiesMinneapolis, MN, USA

**Keywords:** graded response model, multidimensionality, item parameters, sample size, parameter recovery

## Abstract

Likert types of rating scales in which a respondent chooses a response from an ordered set of response options are used to measure a wide variety of psychological, educational, and medical outcome variables. The most appropriate item response theory model for analyzing and scoring these instruments when they provide scores on multiple scales is the multidimensional graded response model (MGRM) A simulation study was conducted to investigate the variables that might affect item parameter recovery for the MGRM. Data were generated based on different sample sizes, test lengths, and scale intercorrelations. Parameter estimates were obtained through the flexMIRT software. The quality of parameter recovery was assessed by the correlation between true and estimated parameters as well as bias and root-mean-square-error. Results indicated that for the vast majority of cases studied a sample size of *N* = 500 provided accurate parameter estimates, except for tests with 240 items when 1000 examinees were necessary to obtain accurate parameter estimates. Increasing sample size beyond *N* = 1000 did not increase the accuracy of MGRM parameter estimates.

## Introduction

A wide variety of psychological, educational, and medical outcome variables are measured using Likert types of rating scales in which a respondent endorses a response from an ordered set of options (e.g., Bjorner et al., 2003; Bolt et al., 2004; Scherbaum et al., 2006). Responses to these types of items have been scored using a number of methods/models (e.g., van der Linden and Hambleton, 1997; Nering and Ostini, 2010), among which the graded response model (GRM; Samejima, 1969) is one of the most popular polytomous item response theory (IRT) models that are able to utilize all the information from each item response in order to better measure people and to create psychometrically sound measuring instruments.

The unidimensional GRM first models, for each item, *K* boundary functions where (*K*+1) is the number of response options. They represent the probability of responding with all options below the boundary vs. all options above the boundary, as a function of the trait level θ. These functions are two-parameter logistic functions, with the item discrimination constant within an item but variable across items:

(1) $$\begin{array}{l} P_{jk}^{*}\left(\theta \right)=\frac{1}{1+\exp\left[-Da_{j}\left(\theta -b_{jk}\right)\right]} \end{array}$$

with *D* = 1.0 or 1.7 and *k* = 1, …, *K*. When *D* = 1.0, the boundary function is the logistic model; when *D* = 1.7, the function is a logistic approximation of the normal ogive model. In Equation (1), *b*_*jk*_ denotes the boundary parameter of the *k*th category of item *j*, which is on the same scale as θ, and *a*_*j*_ is the discrimination parameter for item *j*. Then, as is shown in Equation (2), the probability of responding with a given response option is obtained by subtraction of adjacent boundary functions, with the probability of responding below the first option set to 1.0 and the probability of responding above the highest option set to 0.0:

(2) $$\begin{array}{l} P_{jk}\left(\theta \right)=P_{jk}^{*}\left(\theta \right)-P_{j\left(k+1\right)}^{*}\left(\theta \right) \end{array}$$

Previous research has established guidelines concerning sample sizes needed to accurately estimate item parameters for the unidimensional GRM through simulation studies. Reise and Yu (1990) investigated the parameter recovery under different true θ distributions, true item discrimination distributions, and calibration sample sizes. They concluded that a minimum of 500 calibration examinees was required to achieve adequate item parameter recovery as reflected in correlations between estimated and true discrimination parameters of 0.85 or greater. Recovery of the item boundary parameters was primarily affected by item discriminations, with lower correlations for less discriminating items. Larger sample sizes improved correlational recovery. Both discrimination and boundary parameter recovery, as indexed by the root-mean-squared-error (RMSE), were affected by sample size and item discriminations. In another simulation study, De Ayala (1994) varied sample size and test length. The results from his unidimensional dataset indicated excellent recovery of both the discrimination and boundary parameters (*r* > 0.89 and generally well above 0.90) with 375 examinees for 15-item scales and 750 examinees for 30-item scales.

Many of the instruments that use the GRM include multiple scales measuring different constructs or different aspects of the same construct (e.g., Zickar and Robie, 1999; Fraley et al., 2000; Fletcher and Hattie, 2004; Zagorsek et al., 2006; Walton et al., 2008; Pilkonis et al., 2014). When modeling responses from multidimensional scales using ordered response category items, the multidimensional version of the GRM is the theoretically correct choice. In these studies, however, the unidimensional version of the GRM was used to derive IRT scores for each scale separately, even though the scales were frequently correlated. Such analysis generally fails to capture the association between the constructs in question, and may lead to other undesirable issues, such as model-data misfit. In addition, the use of univariate IRT models in instruments that have an underlying multivariate structure results in a loss of information in the scale scores, yielding scores that are less precise than they could be if an appropriate multivariate model had been applied. De Ayala's study (1994), which also examined the effect of estimating multidimensional data with the unidimensional GRM, showed substantial reductions in the recovery of the discrimination parameters when the incorrect model was estimated (De Ayala, 1994).

The increase in computing power of personal computers and the development of advanced psychometric software—for example, the “*mirt*” package in *R* (R Core Team, 2015), *Mplus* (Muthén and Muthén, 1998–2012), and *flexMIRT* (Cai, 2013)—now make it possible to calibrate complex IRT models on relatively long tests with large samples. A recent study (Thomas et al., 2013) used the multidimensional GRM (MGRM) to score the responses to the Penn Face Memory Test, a measure of visual episodic memory. Hsieh et al. (2010) combined the MGRM with latent growth curve analysis in modeling the longitudinal association between adolescents' social isolation and delinquency. Forero et al. (2013) applied MGRM scoring to the two-dimensional Short Form-12 questionnaire, a widely used measure of physical and mental health status and change. However, these studies utilized preexisting large-scale survey data and did not address the issue of whether the parameters could be accurately estimated given their research sample and the characteristics of their measuring scales. Researchers usually report only model-fit indices, as it is impossible to verify if the model parameters are estimated satisfactorily based on real data.

In addition, there are some concerns and confusion in the literature regarding the appropriate conditions under which such models can be implemented. For example, Ferrando and Chico (2001) chose not to use the MGRM in their data analysis based on a sample of 448 examinees for a 20-item scale because they believed that the model required large sample sizes to obtain accurate parameter estimates (p. 1127). Forero et al. (2009) and Forero and Maydeu-Olivares (2009) reported two extensive simulation studies that examined parameter recovery for multidimensional GRM data using methods implemented in a factor analytic framework. The first study compared diagonally weighted least squares and unweighted least squares estimation methods and the second compared full information maximum likelihood factor analysis and categorical item factor analysis. Their data for both studies were simulated across 324 conditions, varying samples sizes (200, 500, and 2000), test length (9, 21, and 42 items), and factor loadings (0.4, 0.6, 0.8, roughly equivalent to low, medium, and high discriminations). They also included conditions with three latent traits based on items with five response options. They concluded that in most cases 500 examinees were sufficient to adequately estimate item parameters with some of the methods they examined. The usefulness of their results is limited, however, because they assumed that the three traits were uncorrelated, which is rare with typical rating scales used in psychological research and applications. Their results are also limited to the methods they examined in the study, and they did not consider instruments with more than 42 items.

### Purpose

The present study was designed to examine the sample size requirements for obtaining adequate model calibration under the MGRM using standard IRT estimation procedures under a set of realistic conditions to assist researchers in making informed decisions on research design and scale construction when using the MGRM in their data collection and analysis, particularly with larger numbers of items than had been investigated previously.

## Methods

The performance of parameter recovery for the MGRM under several conditions was assessed in a simulation study. The multivariate generalization of the two-parameter logistic version of the model in Equation (1) was used. Let, θ be a vector of length *H* representing the latent traits of interest, then

(3) $$\begin{array}{l} P_{jk}^{*}\left(\theta \right)=\frac{1}{1+exp\left[-D\sum_{h=1}^{H}\left[a_{jh}\left(\theta_{h}-b_{jk}\right)\right]\right]} \end{array}$$

where *h* = 1, 2, …, *H*, and *a*_*jh*_ are the item discrimination parameters on the *h*th dimension of item *j*. *D* was set to 1. Similar to the unidimensional case, $P_{j0}^{*}\left(\theta \right)\equiv 1$ and $P_{j\left(K+1\right)}^{*}\left(\theta \right)\equiv 0$.

### Manipulated variables

Previous research (e.g., Reise and Yu, 1990) has investigated the effects of sample size and test length for the unidimensional GRM. In the multidimensional case, the intercorrelations between dimensions can also affect parameter recovery (De Ayala, 1994). Therefore, three variables were manipulated: (1) test length: *L* = 30, 90, and 240 items; (2) sample size: *N* = 500, 1000, 1500, and 2000; (3) intercorrelation between dimensions: *r* = 0.2, 0.5, and 0.7. The three levels of test length were chosen to simulate different measuring instruments, from short paper-and-pencil personality inventories to an item bank for use in a computerized adaptive test. All the factors were fully crossed, resulting in a total of 3 × 4 × 3 = 36 simulation conditions.

### Items

The three-dimensional graded response model with four response categories for all items was used in this study. The *a*_*jh*_s were randomly sampled from U[1.1, 2.8]. A simple structure was assumed so that for each item only one of the three discrimination parameters was non-zero, and every dimension was represented by an equal number of items. *b*_*jk*_s ranged from [−2, 2] (De Ayala, 1994), and each was uniformly distributed along an equidistant interval within this range for each item. Thus, the three boundary parameters were sampled randomly from U[−2, −0.67], U[−0.67, 0.67], and U[0.67, 2], respectively. To avoid sparseness of the response matrix, only adjacent boundary parameters with distance of at least 0.5 apart were retained.

### Simulees

The latent trait vector θ of an examinee was generated from a multivariate normal distribution with a mean vector of 0s and a covariance matrix with 1s along the diagonal. The off-diagonal terms represented the correlation between any two of the marginal distributions and were set to be a known constant specified above. The true item and person parameters were used in Equation (3) to calculate the theoretical probability of endorsing each category of a given item. The probability vector was provided as the parameter of a multinomial distribution for each item and the first random draw from this distribution was used as the response for the item. Data generation was implemented in R statistical programming software (R Core Team, 2015).

### Item parameter estimation

The generated response matrix was then supplied to flexMIRT2 (Cai, 2013) from which item parameter estimates were obtained using the default Expectation Maximization (EM) algorithm. The default Gauss-Hermite quadrature method was invoked to obtain numeric approximation of the multidimensional integrals. Due to multidimensionality and long test length in some of the simulation conditions, the number of integration quadrature points was reduced from the default of 49 to 21, and the range was restricted to -3.5 to 3.5. Parallel processing was also adopted to further speed up the computation.

### Parameter recovery

The estimated parameters were then used to derive indices of parameter recovery. Bias, RMSE, and Pearson correlations were calculated for the three discrimination and the three boundary parameters, respectively, for each replication. Taking the first discrimination parameter as an example, given a certain dimension, the bias was calculated as

(4) $$\begin{array}{l} Bias=\frac{\sum_{j=1}^{L}\left({â}_{j1}-a_{j1}\right)}{L} \end{array}$$

where *L* is the total test length. RMSE was calculated as

(5) $$\begin{array}{l} RMSE=\sqrt{\frac{\sum_{j=1}^{L}{\left({â}_{j1}-a_{j1}\right)}^{2}}{L}} \end{array}$$

The results were further analyzed by a three-way analysis of variance (ANOVA) for each of the three parameter recovery variables. To facilitate comparison of effects and interactions among the manipulated variables, effect size η^2^ was calculated for each recovered item parameter and was defined as

(6) $$\begin{array}{l} \eta^{2}=\frac{{SS}_{\text{between}}}{{SS}_{\text{total}}} \end{array}$$

where *SS*_*between*_ is the sum of squares between effects and *SS*_*total*_ is the total sum of squares of the model.

### Replications

**Figure 1:**
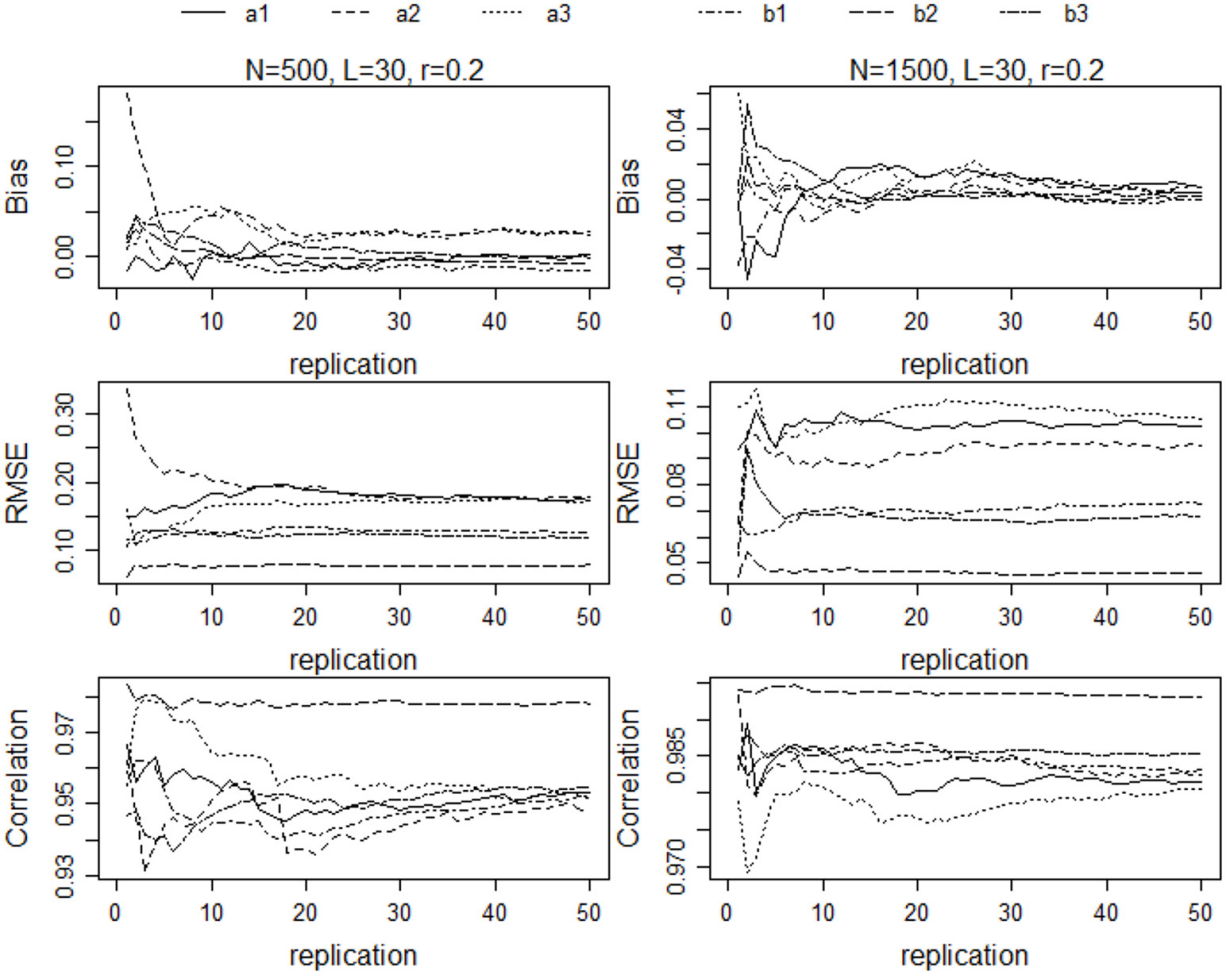
**Bias, RMSE, and correlations of parameter estimates for *N* = 500, *L* = 30, and *r* = 0.2; and *N* = 1500, *L* = 30, and *r* = 0.2**.

To enhance the generality of the results, each of the 36 cells of the completely crossed design was replicated a number of times. To determine the number of replications, a pilot study was conducted in which the simulation procedure was implemented on a selected subset of the 36 conditions for a large number of replications and mean values of the dependent variables were examined as a function of the number of replications. For each replication a new set of θs and item parameters were sampled. To identify an appropriate number of replications for all conditions in the study, the pilot study was implemented for the conditions in which test length ranged from 30 to 90 and sample size from 500 to 2000. Means across each number of replications were then determined and reported for the correlation, bias, and RMSE indices. Figure 1 shows the results for two of the conditions: *L* = 30, *N* = 500, *r* = 0.2; and *L* = 30, *N* = 1500, and *r* = 0.2. As the figures show, for the majority of the *a* and *b* parameters, the recovery indices changed very little after 30 replications. The correlations changed the most from 30 to 50 replications, but the largest change was in the third decimal place. Similar results were obtained for the other conditions in the pilot study. Based on these results, each of the 36 conditions in the study was replicated 30 times.

## Results

Parameter estimation finished normally for all conditions except those with *L* = 240 and *N* = 500, for which the standard errors of estimation for item parameters were unusually large. The results for the three conditions that combined these values of *L* and *N* were, therefore, not included in the subsequent analyses. The longest run time was for the *N* = 2000 and *L* = 240 condition, which ranged from approximately 1800 s to 5000 s per replication.

### Bias

Table 1 shows η^2^-values for each of the main effects and the two- and three-way interactions. Only test length had η^2^s greater than 0.05. For the discriminations (*a*) η^2^s ranged from 0.268 to 0.307; for the *b* parameters, η^2^was 0.236 for *b*_3_ and 0.244 for *b*_1_, but 0.0 for *b*_2._ η^2^s for all other main effects and all interactions were near 0.0.

**Table 1:** Effect sizes (η^2^)^*^ of main effects and interactions on bias of estimates.

**Source of Variation**	***a*_1_**	***a*_2_**	***a*_3_**	***b*_1_**	***b*_2_**	***b*_3_**
Test Length	0.281	0.307	0.268	0.244	0.000	0.236
Sample Size	0.005	0.016	0.014	0.003	0.001	0.000
Correlation	0.001	0.005	0.001	0.001	0.000	0.003
Test Length × Sample Size	0.007	0.008	0.004	0.015	0.003	0.002
Test Length × Correlation	0.008	0.010	0.004	0.001	0.004	0.014
Sample Size × Correlation	0.000	0.000	0.001	0.001	0.000	0.000
Test Length × Sample Size × Correlation	0.000	0.002	0.000	0.001	0.001	0.000
Residual	0.698	0.652	0.708	0.734	0.991	0.746

* *All effect size η^2^ in this and subsequent tables was defined as*
$$\begin{array}{l} \eta^{2}=\frac{{SS}_{between}}{{SS}_{total}} \end{array}$$
*where SS_between_ is the sum of squares between effects and SS_total_ is the total sum of squares of the model*.

**Figure 2:**
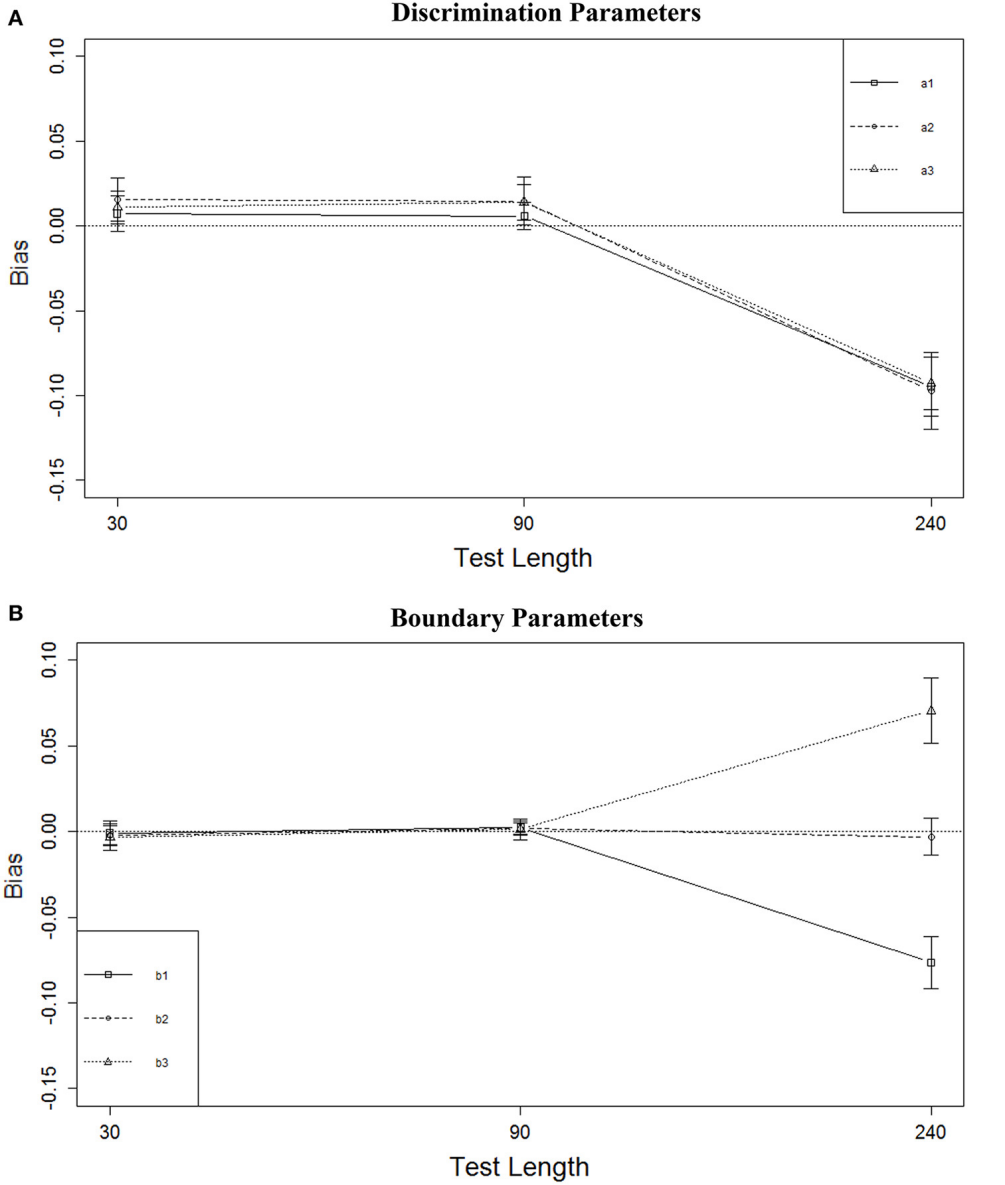
**Bias of parameter estimates for three test lengths. (A)** Discrimination parameters. **(B)** Boundary parameters.

Figure 2A shows the marginal means of bias (aggregated across all other conditions) for the discrimination parameters for the different test lengths; Figure 2B shows similar results for the boundary parameters (vertical lines in these figures represent 1 standard deviation of the means across the 12 conditions for *L* = 30 and *L* = 90, and nine conditions for *L* = 240). These results indicate that the test length effect was mainly due to relatively large bias associated with the *L* = 240 condition, for both the *a* and *b* parameters. The average biases in the other test length conditions were very similar and all reasonably small. Increasing the test length from 30 to 90 did not change the bias for either the *a* or *b* parameters. It was noteworthy (Figure 2B) that there was a strong relationship between bias and the positions of the boundary parameters— *b*_1_s were substantially underestimated while *b*_3_s were overestimated.

### RMSE

Table 2 shows η^2^-values for each of the main effects and two-and three-way interactions with RMSE as the dependent variable. η^2^s for test length were low and near 0.05 for the discrimination parameters but between 0.180 and 0.226 for the boundary parameters. For sample size, η^2^s ranged from 0.295 to 0.336 for the discrimination parameters and 0.215 to 0.266 for the boundary parameters. All other η^2^s were 0.03 or less with the exception of the test length × sample size interaction for *a*_2_, for which η^2^ = 0.049.

**Table 2:** **Effect sizes (η^2^) for main effects and interactions on RMSE of estimates**.

**Source of Variation**	***a*_1_**	***a*_2_**	***a*_3_**	***b*_1_**	***b*_2_**	***b*_3_**
Test Length	0.055	0.048	0.049	0.226	0.218	0.180
Sample Size	0.295	0.335	0.336	0.215	0.266	0.244
Correlation	0.005	0.002	0.002	0.000	0.001	0.005
Test Length × Sample Size	0.030	0.049	0.024	0.030	0.008	0.012
Test Length × Correlation	0.006	0.020	0.008	0.004	0.000	0.006
Sample Size × Correlation	0.000	0.000	0.001	0.001	0.000	0.000
Test Length × Sample Size × Correlation	0.000	0.001	0.000	0.001	0.001	0.000
Residual	0.609	0.545	0.579	0.524	0.506	0.552

**Figure 3:**
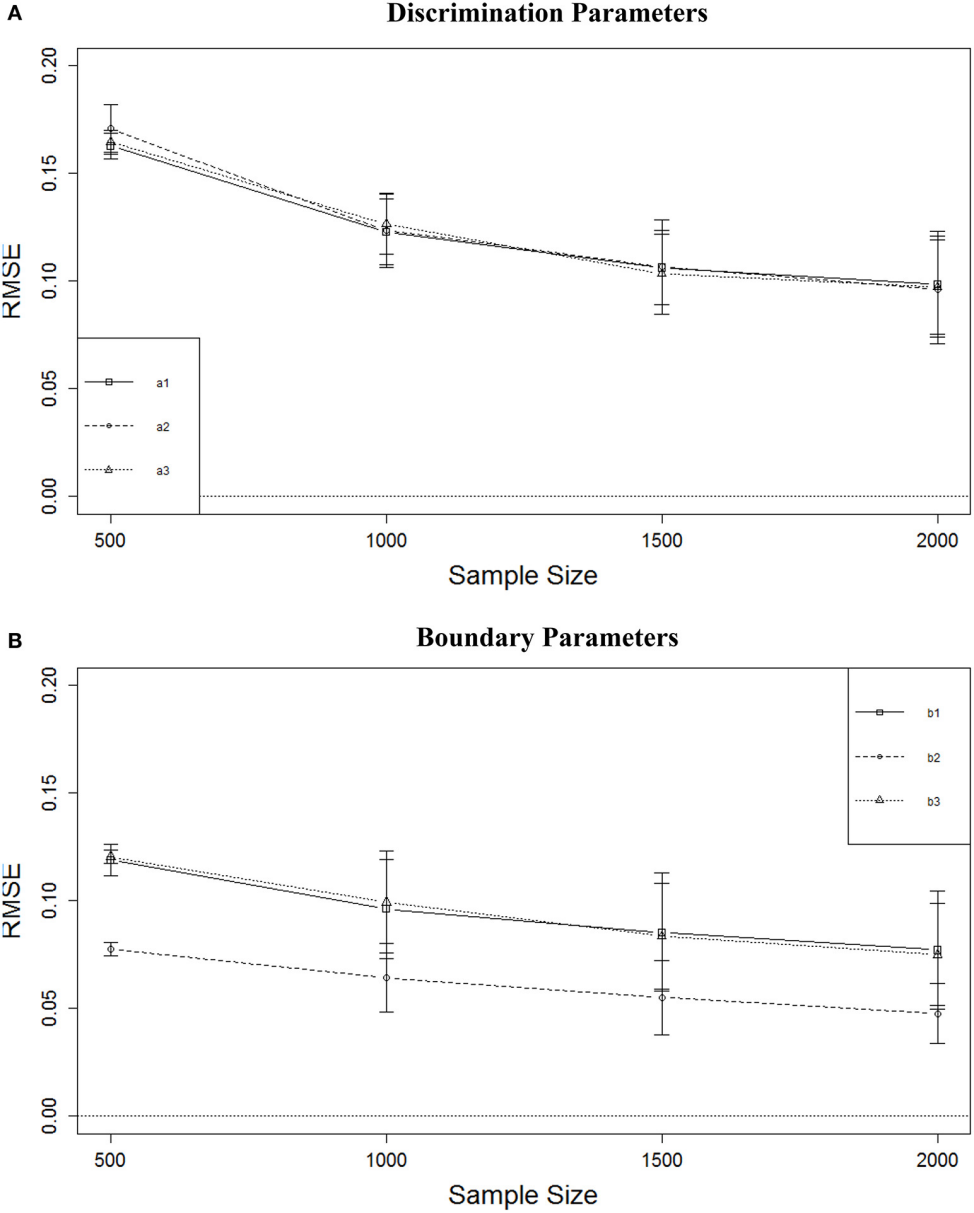
**RMSE of parameter estimates for four sample sizes. (A)** Discrimination parameters. **(B)** Boundary parameters.

Figure 3A shows that the RMSE of the *a* parameter estimates decreased as sample size increased. with the largest decrease between *N* = 500 and *N* = 1000. The average decrease in RMSE from *N* = 1000 to *N* = 1500 was about 0.02. There was almost no decrease in average RMSE from *N* = 1500 to *N* = 2000. A similar result was observed for the boundary parameters (Figure 3B). The largest decrease was observed from *N* = 500 to *N* = 1000, but that decrease was only approximately 0.02. Although RMSE continued to decrease with larger sample sizes, the rate of decrease was slower and the magnitudes of the decreases were very small. Figure 3B also shows that the RMSE for the middle boundary (*b*_2_) was consistently smaller than that of the two more extreme boundaries.

**Figure 4:**
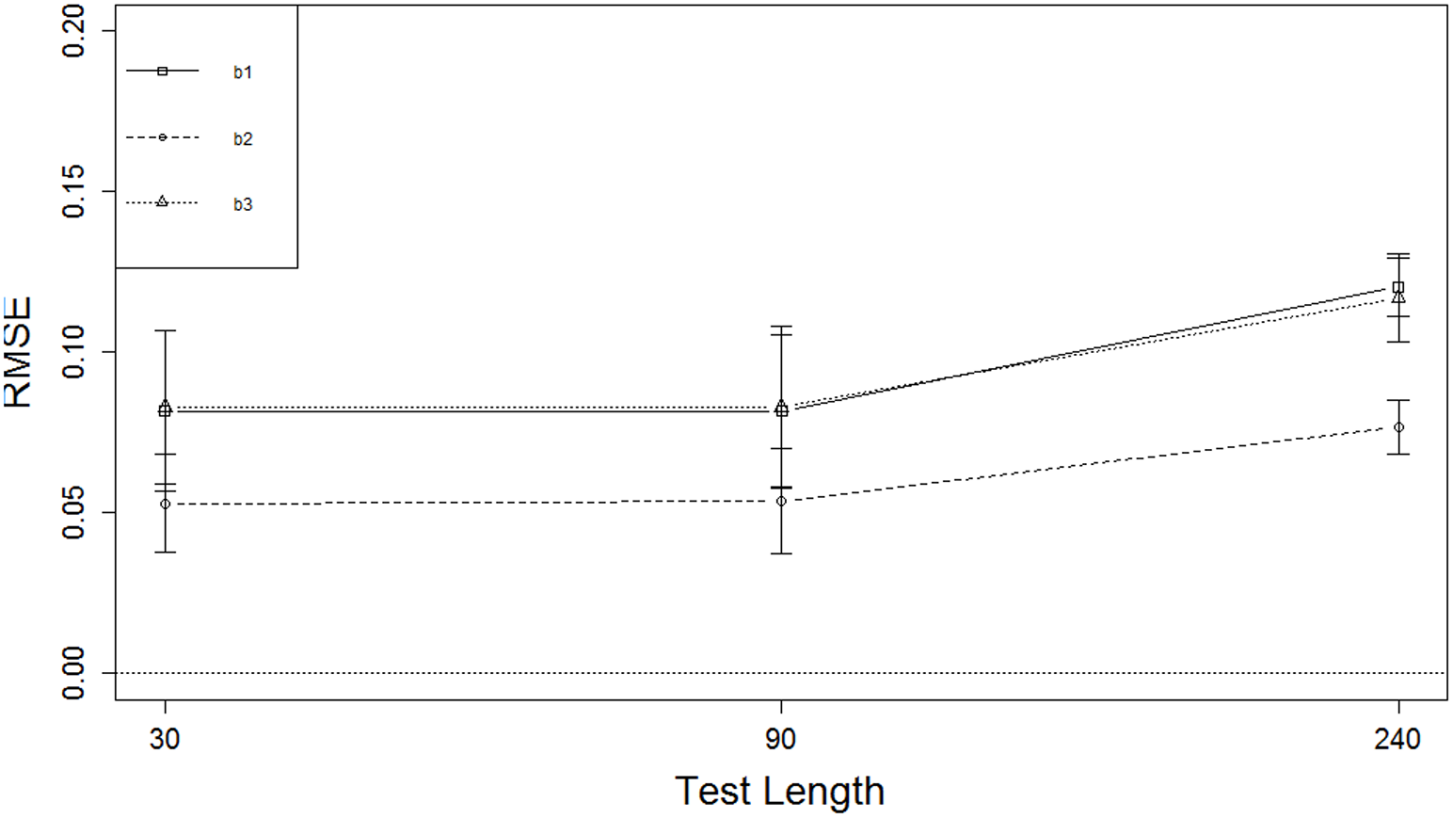
**RMSE of boundary parameters for three test lengths**.

Figure 4 displays marginal means (and SDs) of RMSE for the test length effect. Shorter test length resulted in smaller RMSE for the boundary parameters but not for the discrimination parameters. There was no change in the boundary parameters as test length increased from *L* = 30 to *L* = 90, but RMSE increased from *L* = 90 to *L* = 240 by about 0.04 for the two extreme boundaries and about 0.02 for the middle boundary, which was better recovered than the other two. In general, the boundary parameters were better recovered than the discrimination parameters, and the boundary parameters in the middle were better recovered than those at the ends. The marginal means for RMSE of the discrimination parameters were below 0.20, and those of the boundary parameters were below 0.125, indicating that overall the item parameters were recovered reasonably well.

### Correlation

**Figure 5:**
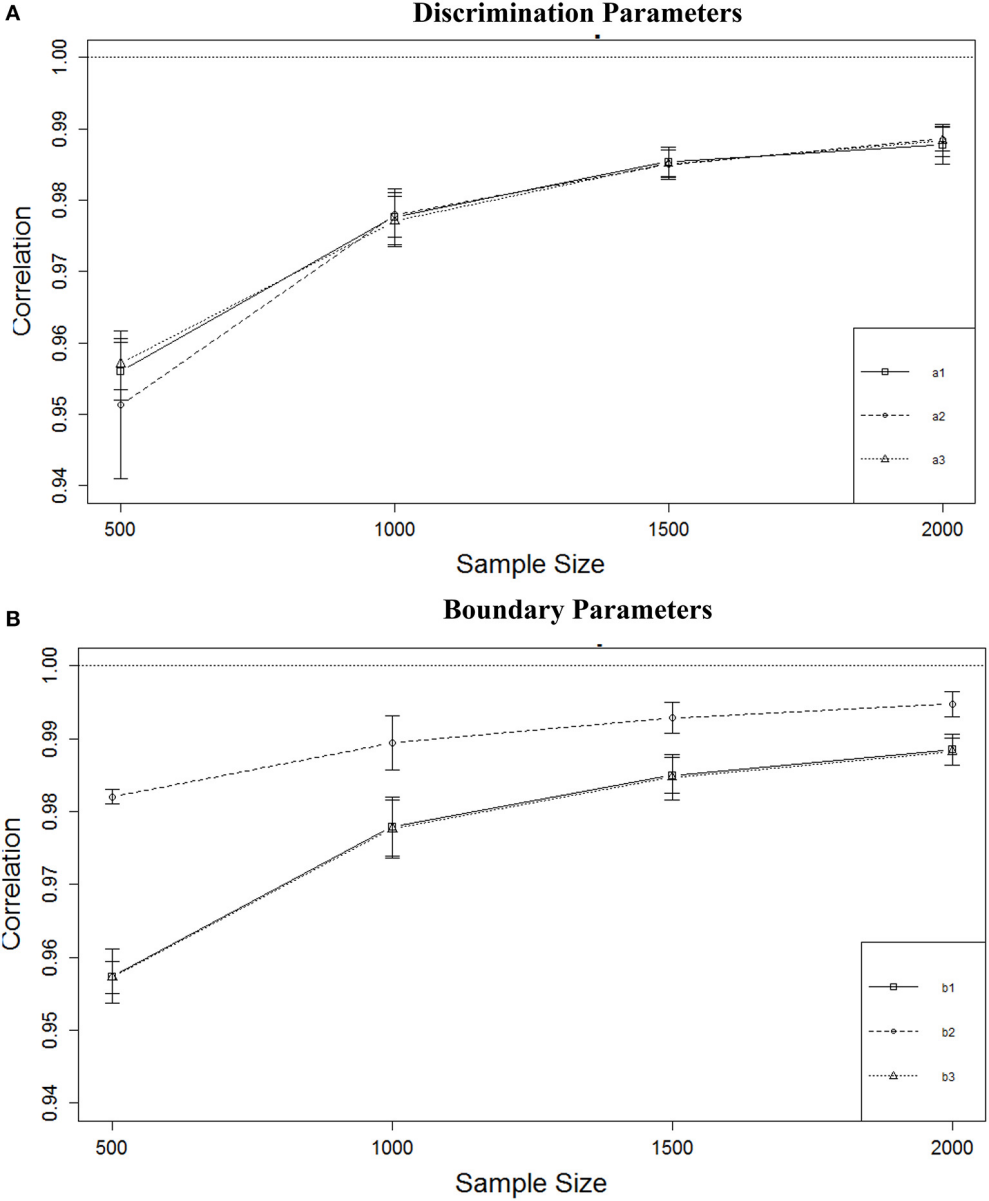
**Correlation between true and estimated parameters for four sample sizes. (A)** Discrimination parameters. **(B)** Boundary parameters.

Table 3 shows that the correlations between true and estimated parameters were affected only by sample size, and the effect was particularly strong for boundary parameters with η^2^s ranging from 0.459 to 0.519 vs. 0.276 to 0.367 for the discrimination parameters. As can be seen from Figure 5A, increasing sample size from 500 to 1000 improved the correlation for the *a* parameters from a mean of about *r* = 0.95 to about *r* = 0.98, but not much gain could be achieved by increasing sample size even further. For the *b* parameters (Figure 5B) the same increase was observed for the *b*_1_ and *b*_3_ parameters and a smaller increase was observed for the *b*_2_ parameter. As was the case with RMSE, the recovery for the middle boundary parameter was better than the others. Both *a* and *b* parameters were recovered with a minimum mean *r* = 0.95 regardless of sample size or number of items.

**Table 3:** **Effect sizes (η^2^) for main effects and interactions on correlation between true and estimated parameters**.

**Source of Variation**	***a*_1_**	***a*_2_**	***a*_3_**	***b*_1_**	***b*_2_**	***b*_3_**
Test Length	0.031	0.024	0.027	0.001	0.029	0.001
Sample Size	0.345	0.276	0.367	0.511	0.459	0.519
Correlation	0.000	0.002	0.000	0.016	0.025	0.009
Test Length × Sample Size	0.009	0.028	0.009	0.011	0.001	0.013
Test Length × Correlation	0.001	0.001	0.001	0.004	0.021	0.005
Sample Size × Correlation	0.000	0.001	0.000	0.003	0.002	0.000
Test Length × Sample Size × Correlation	0.000	0.002	0.001	0.000	0.001	0.001
Residual	0.614	0.665	0.595	0.456	0.462	0.452

## Discussion and conclusions

Three factors that might affect the recovery of MGRM item parameters—sample size, test length, and scale correlations—were varied in a completely crossed simulation design. Effects were evaluated using bias, RMSE, and correlations of the parameter estimates as they recovered true item discrimination and boundary parameters. Results based on using a marginal maximum likelihood IRT estimation procedure, and summarized within an ANOVA framework, indicated that there were no important two-way or three-way interactions for any of the dependent variables—all interactions across the three dependent variables accounted for less than 5% of the variance as reflected in η^2^ values. These results contrast sharply with those of Forero et al. (2009) who observed substantial complex interactions among their independent variables when IRT parameters for the MGRM were estimated within a factor analytic framework using two different least-squares estimation methods.

Of the three manipulated variables, sample size resulted in η^2^ values between 0.276 and 0.367 for recovery of the *a* parameters as indexed by both RMSE and correlations, and between 0.215 and 0.519 for the *b* parameters. Bias of the parameter estimates was not affected by sample size. For both the *a* and *b* parameters, RMSE decreased as sample size increased, with the largest decrease from *N* = 500 to *N* = 1000. Yet for the *a* parameter, that largest decrease was from mean RMSE = 0.17 to mean RMSE = 0.125; for the *b* parameters, the decrease in mean RMSE was 0.02 or less as sample size increased from 500 to 1000. Results for RMSE of the *b* parameters indicated that the two extreme *b* parameters (*b*_1_ and *b*_3_) were better estimated than the center boundary parameter (*b*_3_). This phenomenon is consistent with the well-documented outward bias of maximum likelihood estimators (e.g., Warm, 1989). Although changes in sample size resulted in the largest η^2^ values observed in the analyses for the correlation dependent variable, and correlations increased with sample size, increases in correlations were minimal, with mean correlations varying from about 0.952 to 0.992; these values are much higher than those obtained by Reise and Yu (1990) in their examination of parameter recovery for the unidimensional GRM Again, the *b*_2_ parameters were better recovered than the *b*_1_ or *b*_3_ parameters regardless of sample size. Sample size did not result in any change in bias because all bias values were close to 0.0, even with sample sizes as small as 500.

Variation in test length resulted in relatively large η^2^ values for both the *a* and *b* parameters as reflected in bias and RMSE for the *b* parameters. Correlations were not affected by test length for either *a* or *b*. Mean bias for the *a* parameters was essentially zero for 30-item tests with 10 items per scale and 90-item tests with 30 items per scale. For 240-item tests (80 items per scale) bias increased to a mean of about -0.10. A similar effect was observed for the *b* parameters, except that the observed mean bias of about 0.07 was negative for *b*_1_ and positive for *b*_3_, whereas *b*_2_ was unbiased. This latter result is, again, likely due to the outward bias of maximum likelihood estimators. Overall, however, the degree of bias observed for both parameters was minimal.

The results demonstrated that scale intercorrelations, which were varied from low to high at three levels, had no effect on any of the three parameter recovery indices. All η^2^-values for scale intercorrelations were very near zero.

The present study differed from the two studies by Forero et al. (2009) in several repects: (1) the correlations among dimensions were varied at 0.2, 0.5, or 0.7, whereas the Ferero studies held the scale correlation at zero; (2) the present study considered much longer test length. Even though Forero et al. argued that short questionnaires are frequently encountered in behavioral research within medical settings, the present study examined the sample size requirements for accurately calibrating much larger numbers of items. This choice was made because large numbers of items are needed to form item banks to facilitate computerized adaptive testing. Finally, the Forero studies estimated the model using factor analysis methods, which result in a different parameterization than the traditional IRT parameterization applied in the present study. Nevertheless, with regard to the major common element among the three studies, the results agreed that a sample size as small as *N* = 500 is sufficient to recover all model parameters adequately—even with tests that had three scales of 80 items each—and sample size larger than 1000 does not provide any visible benefit for parameter recovery. The exception to *N* = 500 occurred in the present study using the traditional IRT parameterization was 240-item tests. In that case, the standard errors of the parameter estimates were large, indicating inaccurate estimates. Under these circumstances, *N* = 1000 was required to obtain estimates with useful standard errors. Using the factor analysis parameterization, Forero et al. had estimation failures under several other conditions with *N* = 500 but with much shorter tests.

Level of correlation in this study did not show any effect on parameter recovery, primarily because only simple structure was considered in the design. Usually higher correlation is preferred when estimating person parameters because the shared information among correlated dimensions will help reduce measurement errors of θ (e.g., Wang, 2015). However, higher correlation might also impose estimation difficulty for item parameter recovery possibly due to multicollinearity (e.g., Babcock, 2011; Wang and Nydick, 2014). This phenomenon is especially salient for within-item multidimensionality. Therefore, it would be interesting to extend the present research to complex test structures.

This research can also be generalized in many other directions. For instance, only the EM algorithm was evaluated in the present study. Note that in flexMIRT, either EM or the Metropolis-Hastings Robbins-Monro (MH-RM) algorithm is implemented, with the latter allowing users to deal effectively with higher-dimensional models (i.e., models with more than three latent variables). The major difference between the two is their performance on models with high dimensions, and the difference has more to do with speed than accuracy (Cai, 2010). EM was used because it is currently the most commonly used algorithm in IRT research and the model did not contain too many dimensions. In addition, it was possible to achieve acceptable computing speed by modifying a few options under EM. A future study can be conducted to compare different available estimation methods, such as the Markov Chain Monte Carlo algorithm, among others.

Second, all latent traits were assumed to follow a normal distribution. However, it is highly possible, especially in medical or clinical data, that certain latent traits in a general population follow a skewed distribution. Consequently, it would be appropriate to examine the robustness of parameter recovery for the current MGRM model in the presence of non-normal θs and, if the estimation procedures are not robust to non-normality, develop a new semi-parametric MGRM model to account for non-normality of θs in the model.

## Author contributions

SJ is the first author of the manuscript. He was in charge of running the simulation study and drafting the introduction, method, and results section of the manuscript. CW is the second author and corresponding author. She contributed to the original idea of the study and literature review. She was also in charge of designing/refining the simulation study as well as writing the discussion section of the paper. DW is the last author and he oversaw the entire project. He also revised and edited the entire manuscript.

### Conflict of interest statement

The authors declare that the research was conducted in the absence of any commercial or financial relationships that could be construed as a potential conflict of interest.

## References

[B1] Babcock B. (2011). Estimating a noncompensatory IRT model using Metropolis within Gibbs sampling. Appl. Psychol. Meas. 35, 317–329. 10.1177/0146621610392366

[B2] Bjorner J. B. Kosinski M. Ware J. E. Jr.. (2003). Calibration of an item pool for assessing the burden of headaches: an application of item response theory to the Headache Impact Test (HIT™). Qual. Life Res. 12, 913–933. 10.1023/A:1026163113446 14651412

[B3] Bolt D. M. Hare R. D. Vitale J. E. Newman J. P. (2004). A multigroup item response theory analysis of the psychopathy checklist–revised. Psychol. Assess. 16, 155–168. 10.1037/1040-3590.16.2.155 15222812

[B4] Cai L. (2010). High-dimensional exploratory item factor analysis by a Metropolis-Hastings Robbins-Monro algorithm. Psychometrika 75, 33–57. 10.1007/s11336-009-9136-x

[B5] Cai L. (2013). flexMIRT: A Numerical Engine for Flexible Multilevel Multidimensional Item Analysis and Test Scoring (Version 2.0) [Computer software]. Chapel Hill, NC: Vector Psychometric Group.

[B6] De Ayala R. J. (1994). The influence of multidimensionality on the graded response model. Appl. Psychol. Meas. 18, 155–170. 10.1177/014662169401800205

[B7] Ferrando P. J. Chico E. (2001). The construct of sensation seeking as measured by Zuckerman's SSS-V and Arnett's AISS: a structural equation model. Pers. Individ. Diff. 31, 1121–1133. 10.1016/S0191-8869(00)00208-7

[B8] Fletcher R. B. Hattie J. A. (2004). An examination of the psychometric properties of the physical self-description questionnaire using a polytomous item response model. Psychol. Sport Exerc. 5, 423–446. 10.1016/S1469-0292(03)00036-0

[B9] Forero C. G. Maydeu-Olivares A. (2009). Estimation of IRT graded response models: limited versus full information methods. Psychol. Methods 14, 275. 10.1037/a0015825 19719362

[B10] Forero C. G. Maydeu-Olivares A. Gallardo-Pujol D. (2009). Factor analysis with ordinal indicators: a monte carlo study comparing DWLS and ULS estimation. Struct. Equat. Model. 16, 625–641. 10.1080/10705510903203573

[B11] Forero C. G. Vilagut G. Adroher N. D. Alonso J. (2013). Multidimensional item response theory models yielded good fit and reliable scores for the Short Form-12 questionnaire. J. Clin. Epidemiol. 66, 790–801. 10.1016/j.jclinepi.2013.02.007 23707080

[B12] Fraley R. C. Waller N. G. Brennan K. A. (2000). An item response theory analysis of self-report measures of adult attachment. J. Pers. Soc. Psychol. 78, 350–365. 10.1037/0022-3514.78.2.350 10707340

[B13] Hsieh C.-A. von Eye A. A. Maier K. S. (2010). Using a multivariate multilevel polytomous item response theory model to study parallel processes of change: the dynamic association between adolescents' aocial isolation and engagement with delinquent peers in the National Youth Survey. Multivariate Behav. Res. 45, 508–552. 10.1080/00273171.2010.483387 26760491

[B14] Muthén L. K. Muthén B. O. (1998–2012). Mplus User's Guide 7th Edn. Los Angeles, CA: Author.

[B15] Nering M. L. Ostini R. (Eds.). (2010). Handbook of Polytomous Item Response Theory Models. New York, NY: Routledge.

[B16] Pilkonis P. A. Kim Y. Yu L. Morse J. Q. (2014). Adult Attachment Ratings (AAR): an item response theory analysis. J. Pers. Assess. 96, 417–425. 10.1080/00223891.2013.832261 24033268 PMC3954965

[B17] R Core Team (2015). R: A Language and Environment for Statistical Computing. R Foundation for Statistical Computing. Vienna, Austria Available online at: http://www.R-project.org/

[B18] Reise S. P. Yu J. (1990). Parameter recovery in the graded response model using MULTILOG. J. Educ. Meas. 27, 133–144. 10.1111/j.1745-3984.1990.tb00738.x

[B19] Samejima F. (1969). Estimation of Latent Ability Using a Response Pattern of Graded Scores (Psychometric Monograph No. 17). Richmond, VA: Psychometric Society Available online at: http://www.psychometrika.org/journal/online/MN17.pdf

[B20] Scherbaum C. A. Cohen-Charash Y. Kern M. J. (2006). Measuring general self-efficacy: a comparison of three measures using item response theory. Educ. Psychol. Meas. 66, 1047–1063. 10.1177/0013164406288171

[B21] Thomas M. L. Brown G. G. Gur R. C. Hansen J. A. Nock M. K. Heeringa S.. (2013). Parallel psychometric and cognitive modeling analyses of the Penn face memory test in the army study to assess risk and resilience in service members. J. Clin. Exp. Neuropsychol. 35, 225–245. 10.1080/13803395.2012.762974 23383967 PMC3600160

[B22] van der Linden W. J. Hambleton R. K. (Eds.). (1997). Handbook of Modern Item Response Theory. New York, NY: Springer.

[B23] Walton K. E. Roberts B. W. Krueger R. F. Blonigen D. M. Hicks B. M. (2008). Capturing abnormal personality with normal personality inventories: an item response theory approach. J. Pers. 76, 1623–1648. 10.1111/j.1467-6494.2008.00533.x 19012660 PMC2802070

[B24] Wang C. Nydick S. W. (2014). Comparing two algorithms for calibrating the restricted non-compensatory multidimensional IRT Model. Appl. Psychol. Meas. 39, 119–134. 10.1177/0146621614545983 PMC5978509 29880997

[B25] Wang C. (2015). On latent trait estimation in multidimensional compensatory item response models. Psychometrika 80, 428–449. 10.1007/s11336-013-9399-0 24604245

[B26] Warm T. A. (1989). Weighted likelihood estimation of ability in item response theory. Psychometrika 54, 427–450. 10.1007/BF02294627

[B27] Zagorsek H. Stough S. J. Jaklic M. (2006). Analysis of the reliability of the Leadership Practices Inventory in the item response theory framework. Int. J. Sel. Assess. 14, 180–191. 10.1111/j.1468-2389.2006.00343.x

[B28] Zickar M. J. Robie C. (1999). Modeling faking good on personality items: an item-level analysis. J. Appl. Psychol. 84, 551–563. 10.1037/0021-9010.84.4.551

